# Diagnosis and Surgical Treatment of Idiopathic Primary Sino-Nasal Obstruction in Miniature Horse Breeds: Long-Term Follow-Up of Seven Cases

**DOI:** 10.3389/fvets.2021.680150

**Published:** 2021-07-06

**Authors:** Lieven Vlaminck, Elke Pollaris, Katrien Vanderperren, W. Henry Tremaine, Els Raes

**Affiliations:** ^1^Department of Large Animal Surgery, Anaesthesia and Orthopaedics, Faculty of Veterinary Medicine, Ghent University, Merelbeke, Belgium; ^2^Department of Medical Imaging of Domestic Animals and Orthopedics of Small Animals, Faculty of Veterinary Medicine, Ghent University, Merelbeke, Belgium; ^3^B&W Equine Hospital, Breadstone, United Kingdom

**Keywords:** bilateral sinus disease, single caudally based front-nasal bone flap, miniature horse breed, idiopathic sino-nasal obstruction, idiopathic sinus pathology

## Abstract

Idiopathic sino-nasal obstruction resulting in retention of large amounts of liquid in the paranasal sinus compartments was diagnosed in seven young (2. 2 ± 0.7 years) miniature-breed horses based on clinical, endoscopic, radiographic, and CT scan examinations. The most prevalent clinical signs included decreased or no airflow from the nostril(s) (7/7) and nasal discharge (6/7). The problem presented bilaterally in six of seven cases. An alternative sino-nasal communication was created through bone flap osteotomy surgery and perforation of the ventromedial floor of the dorsal conchae in all cases, followed by fixation of silicone irrigation tubes/Foley catheters in six of seven cases to keep the newly created ostium patent. This resulted in long-term resolution of the problem with good cosmetic appearance in all animals following a median period of 19 months. Premature loss of fixed tubes was reported in three cases.

## Introduction

A variety of sino-nasal pathological conditions in horses have been well-described. Common diseases include primary and secondary sinusitis (1–3), paranasal sinus cysts (4), progressive ethmoid hematoma (5, 6), and more rarely encountered sinus neoplasia (7, 8). A correct diagnosis is straightforward in the majority of cases and based on the patient's medical history, clinical symptoms, and the results of further diagnostic procedures such as endoscopy, radiography, computed tomography (CT), sinoscopy, and bacteriological and/or histological analysis of pre-operative retrieved fluid and tissue samples, respectively. Primary sinusitis can respond well to systemic treatment with broad-spectrum antibiotics. Other pathologies often require surgical interventions such as transnasal sinus approaches or external trephination, sinoscopy, or sinusotomy to restore nasomaxillary mucus drainage or to deal with the encountered pathology.

A seldomly reported sinus pathology in horses is sinus mucocele. Mucoceles are believed to develop following obstruction of the nasomaxillary aperture between sinus and the nasal cavity. Progressive deformation of the surrounding structures is the result of continuous production and accumulation of mucus in the isolated sinus compartments. In humans, the most common causes of occlusion of sinus drainage toward the nose are chronic infection, allergic sino-nasal disease, trauma, and previous surgery (9, 10). In equine veterinary literature, this pathology has not been reported in some larger retrospective case series (3, 11). One older case report briefly mentions mucocele sinusitis in a thoroughbred filly resolved by simple trephination, flushing, and antimicrobial treatment (12). A more recent case report describes the use of a caudally based fronto-nasal bone flap and sino-nasal fenestration to successfully resolve bilateral sinus mucocele in an American miniature horse (13). We describe the clinical symptoms, diagnostic work-up, and long-term follow-up of seven comparable cases of sino-nasal obstruction treated by bone flap surgery and restored drainage toward the nasal cavity.

## Materials and Methods

The medical records of six horses presented to the equine services of the Faculty of Veterinary Medicine at Ghent University between January 2011 and January 2018 where a diagnosis of idiopathic sino-nasal obstruction was made were reviewed. In January 2018, an additional call was distributed to EVDC Eq specialists to contribute comparable cases from their case load that added one extra case (WHT) to the study population.

The main criterion that was used to diagnose sino-nasal obstruction included uni- or bilateral accumulation of fluid within sinus compartments in the absence of any other sinus pathology such as sinusitis, paranasal sinus cyst, progressive ethmoid hematoma, or any kind of neoplasia. All horses received a complete clinical examination with special attention to facial deformities in the area of the frontal and maxillary sinus compartments, nasal discharge, and air flow from both nostrils. Nasal endoscopy was performed if possible, *via* both nostrils. The dentition was orally examined for any signs of associated dental disease. Radiographs were acquired using computed radiography systems (Ghent University: Konica Minolta before 2015, Agfa CR30 after 2015, and Agfa DX-M after 2016; B&W Equine Hospital, Siemens Gantry mounted-Agfa CR/Canon-Eklin DR system). Additional computed tomography imaging was performed under general anesthesia in dorsal recumbency (Lightspeed QX/I33, 4-slice helical scanner at Ghent University; Toshiba Aquilon-B, 16-slice helical scanner at B&W Equine Hospital) (120 kVp, 160 mAs, pitch 0.75, slice thickness 1.25–2.5 mm, matrix 512 ×512, scan FOV between 254 and 500 mm, creating bone and soft tissue algorithm reconstructions at Ghent University and at B&W Equine Hospital). In animals in which i.v. jugular vein contrast medium was administered (cases 1–6), a post-contrast acquisition was obtained 2 min after the start of the contrast injection. Contrast medium (Omnipaque, GE Healthcare) at a dosage of 2 ml/kg bodyweight and standardized concentration (iohexol 300 mgI/ml) was administered as a single bolus by use of a power injector.

Surgical exploration was performed using a single caudally based fronto-nasal bone flap as described by Easley and Freeman (13) for cases with bilateral involvement of sinus compartments, or a standard unilateral fronto-nasal bone flap as described by Freeman et al. for unilateral cases (14). Surgery was performed under general anesthesia following induction of anesthesia and orotracheal intubation. Animals were positioned in sternal recumbency to facilitate access to both sides of the head. More recent cases were treated in the standing animal using continuous rate infusion (CRI) of detomidine and regional maxillary nerve blocks (cases 5 and 6) (15). Once the bone flap was elevated, all liquid contents were removed from the sinus compartments by suction followed by copious lavage with Hartmann's. An alternative sino-nasal communication was created by perforating the medio-ventral wall of the rostral aspect of the dorsal conchal sinus by digital pressure or using a blunt tipped instrument. Using a scalpel blade and/or dissection scissors, the hole was enlarged to reach a minimum size of 2 cm. Diffuse bleeding from the conchal wall margins was stopped by temporary pressure using gauze sponges for 5–10 min. Then, a 1.5-cm outer diameter silicone tube (cases 1–6) or 24F Foley catheter (case 7) was introduced to maintain patency of the fistula allowing ongoing drainage. The distal end of each silicone tube was fixed to the ipsilateral nostril with a simple interrupted suture. The Foley catheter remained in position through inflation of the balloon within the sinus. If continuous significant hemorrhage was observed after release of local pressure, the dorsal conchal sinus was packed with gauze bandages for hemostasis and no tube or catheter was introduced. The distal end of the bandage exited through the nasal cavity and was fixed to the nose. The frontal bone flap was replaced, and the periosteum was sutured using a simple continuous pattern with 3M Vicryl Plus (Ethicon). The skin was closed using stainless steel staples (cases 1–6) or simple interrupted sutures through the skin and periosteum using 3.5 M Monosof (Tyco) (case 7). Post-operative irrigation of the sinuses with saline was performed through pre-drilled trephination holes in cases 1–6. A small stab incision was made centered over the ipsilateral concho-frontal sinus compartment followed by drilling a trephination hole with a 3.2-mm bur. The stab incision was closed with two stainless-steel staples. A 16-gauge needle attached to an infusion line and a 1-L bag of sterile saline was passed through the stab incision and the associated trephine hole to allow daily flushing (once or twice a day) for a variable period (cases 1–6). In case 7, the Foley catheters were used to flush the sinus compartments accordingly. Post-operative antimicrobial treatment consisted of intramuscular administration of procaine benzyl penicillin (20,000 IU/kg once daily) for 2–7 days, sometimes continued for another 2 weeks orally with potentiated sulfonamides (25 mg/kg twice daily) based on each patient's individual clinical progression and the surgeon's preference. NSAIDs were administered orally for 3–7 days. If possible, sino-nasal tubes were kept in place for a period of 3 weeks before removal. Owners were advised to box-rest their horses or have them turned out to pasture until removal of sino-nasal tubes.

Owners were contacted by telephone after a minimum time period of 6 months to obtain long-term follow-up information. They were questioned about the recurrence or persistence of symptoms such as abnormal respiratory noise, nasal discharge, and any health problems that might have occurred following the surgical treatment of their horse. They were also asked for their subjective evaluation of the scar at the level of the surgical incision.

## Results

Table 1 summarizes individual data of the seven animals. All animals included in the study group were mares. Breed distribution consisted of four American miniature horses and three mini-Shetland ponies with a mean age of 2.2 ± 0.7 years and ranging in weight from 64 to 140 kg. Median duration of symptoms before referral to a specialized center was 4 weeks (range 3–36 weeks). Treatment with antibiotics and NSAIDs was given in five of seven animals for variable periods without any improvement of symptoms. On admission, clinical parameters such as body temperature, heart rate, and CRT were within normal limits. Apparent clinical symptoms are summarized in Table 2. Decreased air flow from the nostril(s) was present in all animals although varying in location (uni- or bilateral) and severity. Respiratory rate was elevated in two animals that showed open mouth breathing due to bilateral severe narrowing of nasal passages (cases 1 and 2). This necessitated an emergency tracheotomy in one of them (case 2). The latter horse as well as case 6 produced a snoring expiratory noise. Endoscopy of the upper respiratory tract, performed in six of seven horses (not performed in case 7), confirmed varying degrees of narrowing of nasal passages due to medial displacement of the dorsal concha. In three of six animals (cases 1–3), an 8-mm-diameter flexible endoscope could not be introduced further than 2–3 cm. Nasal discharge was variably unilateral (cases 5 and 6) or bilateral (other cases) and was mucoid without an associated abnormal odor. Two animals with bilateral sinus disease (cases 1 and 4) showed mild, bilateral facial deformity in the region of the frontal bones, which was more pronounced on the right side in case 4. A third animal (case 2) had undergone prior bilateral frontal sinus trephination and flushing, which resulted in unilateral (right) localized wound infection and subsequent edematous swelling in that region. Epiphora was not recorded in any of the animals. Dental examination revealed no oral dental pathology in any patient.

**Table 1:** Summary data for horses undergoing treatment of idiopathic sino-nasal obstruction.

**Horse No**.	**Age (years)**	**Breed**	**Sex**	**Duration (weeks)**	**Uni- or bi-lateral**	**Radiographic views**	**CT scan performed**	**Surgery**	**Follow-up (months)**	Premature loss tube^*^/Foley^**^	**Recurrence**
1	3	AM	F	3	Bilateral	4	Yes	CB-FNBF	88	No^*^	No
2	1.7	S	F	3	Bilateral	4	Yes	CB-FNBF	30	Yes^*^ (right)	No
3	3.1	AM	F	4	Bilateral	None	Yes	CB-FNBF	26	No^*^	No
4	1.7	S	F	3	Bilateral	None	Yes	CB-FNBF	19	No^*^	No
5	2.5	AM	F	9	Right side	2	Yes	Right FNBF	14	No^*^	No
6	1.4	S	F	36	Bilateral	4	No	CB-FNBF	9	Yes^*^ (both)	No
7	Unknown	AM	F	9	Bilateral	None	Yes	CB-FNBF	6	Yes^**^ (both)	No

*AM, American miniature horse; S, mini-Shetland pony; F, female; CB-FNBF, caudally based fronto-nasal bone flap.*

* *silicone tube,*

** *Foley catheter*.

**Table 2:** Clinical signs encountered in seven cases with sino-nasal obstruction in miniature horse breeds.

**Clinical signs**	***n***
Decreased/no air flow from nostril(s)	7
Nasal discharge	6
Deformation frontal bone region	2
Open mouth breathing	2

**Figure 1:**
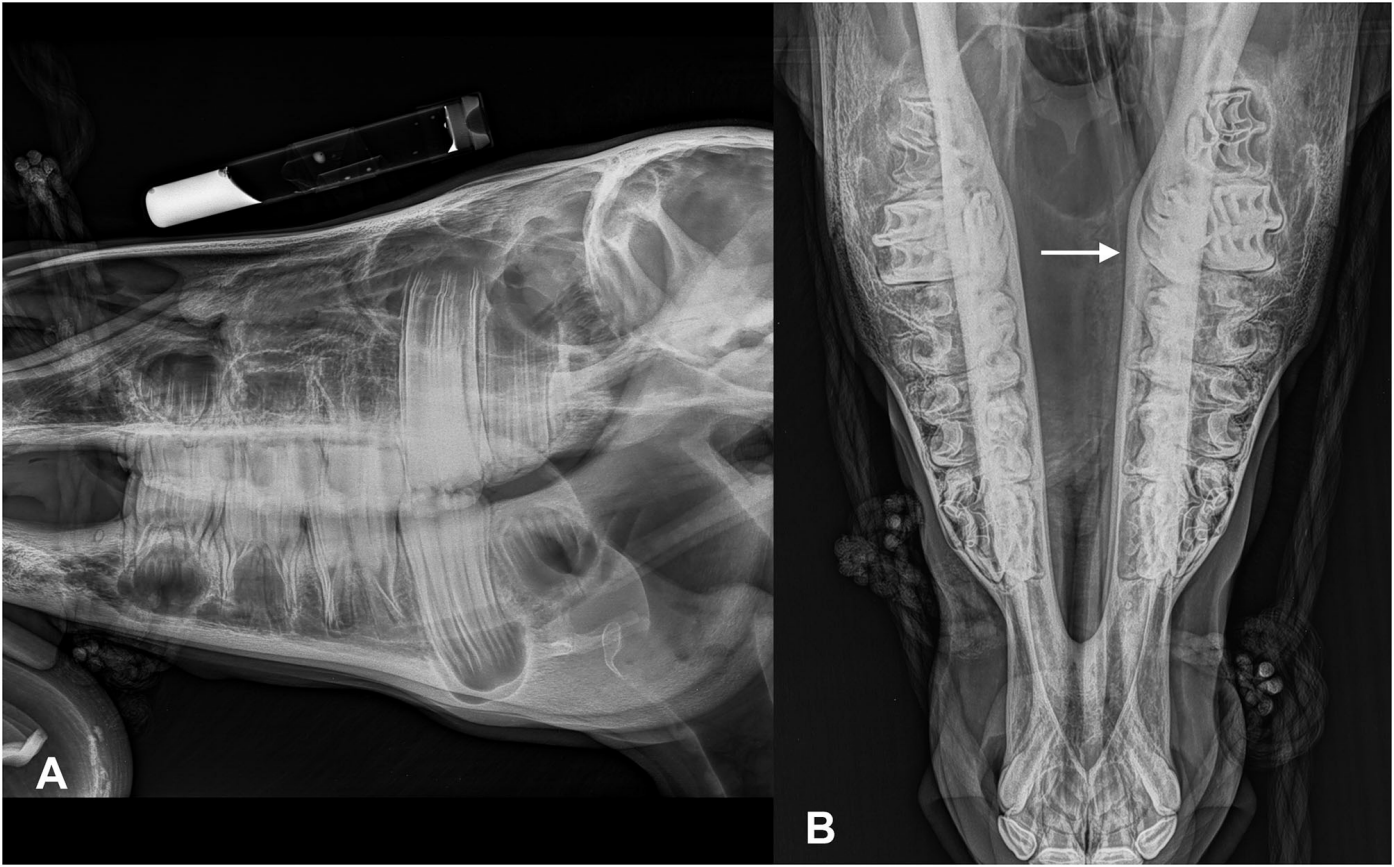
Lateral **(A)** and dorsoventral **(B)** (left in the image is right of the patient) radiographic views of mini-Shetland pony (case 6) presented with bilateral disease. Note that the sinus compartments demonstrate a diffuse soft tissue opacification. The nasal septum is severely deviated toward the left side (white arrow).

Radiographic images were available for four of seven patients. In all patients, laterolateral and dorsoventral views were performed; in three patients, oblique views were also included (right dorsal to left ventral oblique and left dorsal to right ventral oblique). All four patients demonstrated homogeneous diffuse soft tissue opacification in the normally gas-filled frontal and maxillary sinuses. The single patient with unilateral disease (case 5) had only two views included in the study; the dorsoventral projection clearly demonstrated the soft tissue opacification of the right sinus complex. In only one patient (case 6) was there a mass effect noted with marked left-sided deviation of the nasal septum (Figure 1). This patient had bilateral disease; however, this was uncertain based on the radiographic study only.

**Figure 2:**
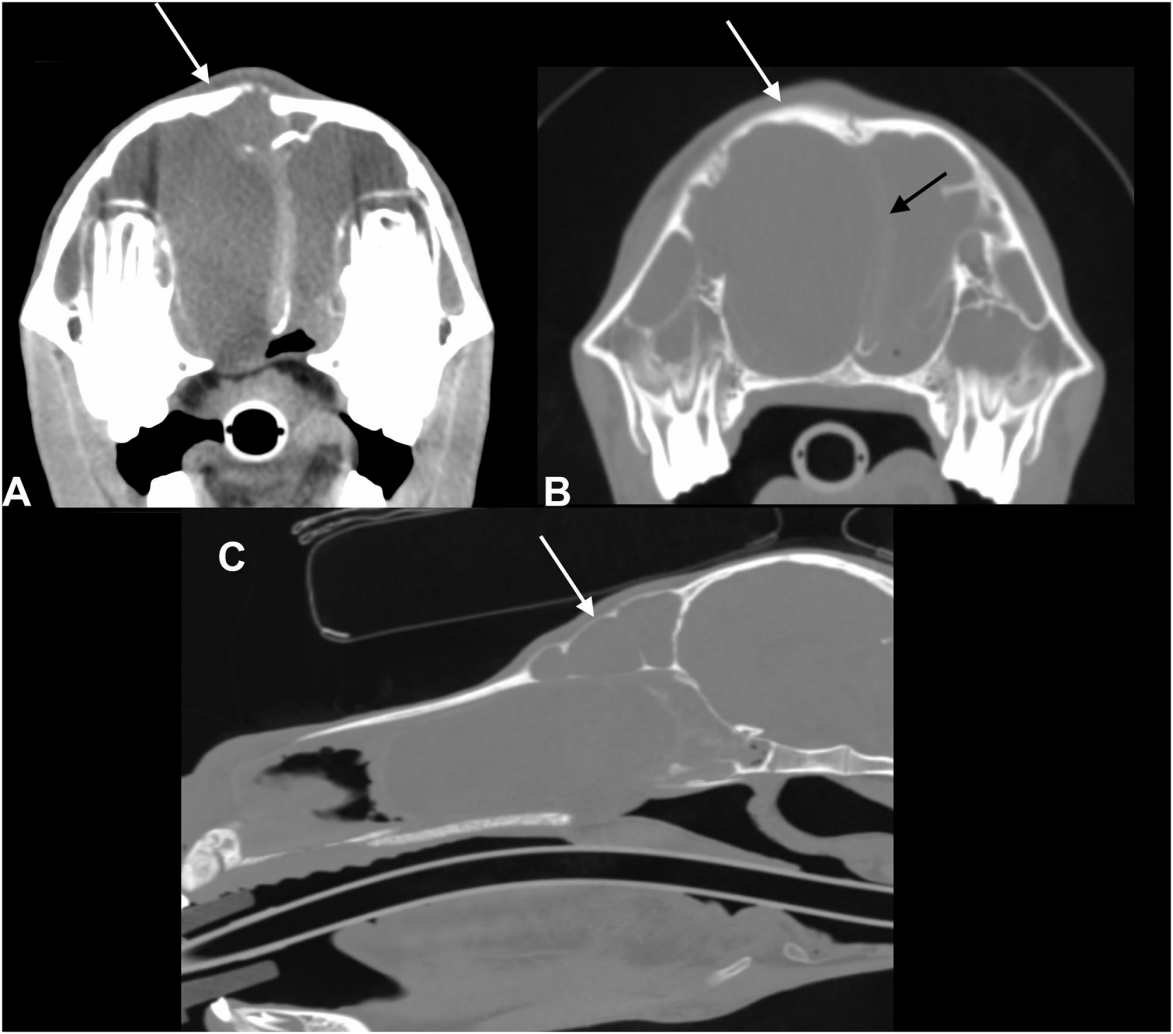
Transverse CT images of a mini-Shetland pony with bilateral sinus disease (case 4) processed with soft tissue **(A)** and bone **(B)** filters (left in the image is right of the patient) and sagittal reformatted image processed with bone filters **(C)**. The white arrows indicate deformation of the frontal bone. The black arrow indicates the leftward shift of the nasal septum. Note the diffuse, homogeneous filling of the left and right sinus compartments with hypoattenuating fluid material; HU of the sinus content in this patient was 17.

A CT study was performed in six of seven patients. In five of six horses, bilateral disease was present, whereas in one patient, the disease was unilateral. In five patients, the affected sinus compartments were almost completely filled with a homogeneous fluid attenuating content. Often a small normal gas attenuating area was only identified at the level of the sphenopalatine sinuses. Hounsfield units (HU) of the fluid-attenuating content varied in five patients from 1 to 25 HU. In case 5, HU were slightly higher and varied from 25 to 35 HU. In another patient (case 2), the sinus compartment was incompletely filled with larger normal gas attenuating areas remaining in the caudal maxillary sinus, frontal sinus, and presence of fluid/gas interfaces in the conchal sinuses. The content was more heterogeneous compared in this case, with dispersed gas attenuating foci within the sinus content. In this patient, trephination osteotomies were present in both the left and right frontal bone. At the level of the right trephination site, there was marked soft tissue swelling present with subcutaneous encapsulated fluid and gas accumulation. There was also a mild to moderate amount of periosteal reaction detected adjacent to the right trephination site. In case 4, there was bony deformation of the skull with focal outward distension of the right frontal bone associated with a moderate amount of ill-defined, periosteal reaction, and thinning of the frontal bone. In this patient, there was also marked septal deviation toward the left side (Figure 2). This patient did not have a radiographic exam before the CT. In all patients, there was marked distension of the conchal sinuses resulting in narrowing of the nasal passages. Deviation of the nasal septum was noted in five patients, in all cases away from the sinus compartments that were most severely affected. Case 1 did not show deviation of the septum as left and right sinus compartments were equally affected. The dentition showed changes consistent with physiological age-related developmental changes that in miniature breeds can be highly variable between individuals and compared to typical changes of larger breeds. However, specific changes pathognomonic for acquired dental pathology were not recorded.

**Figure 3:**
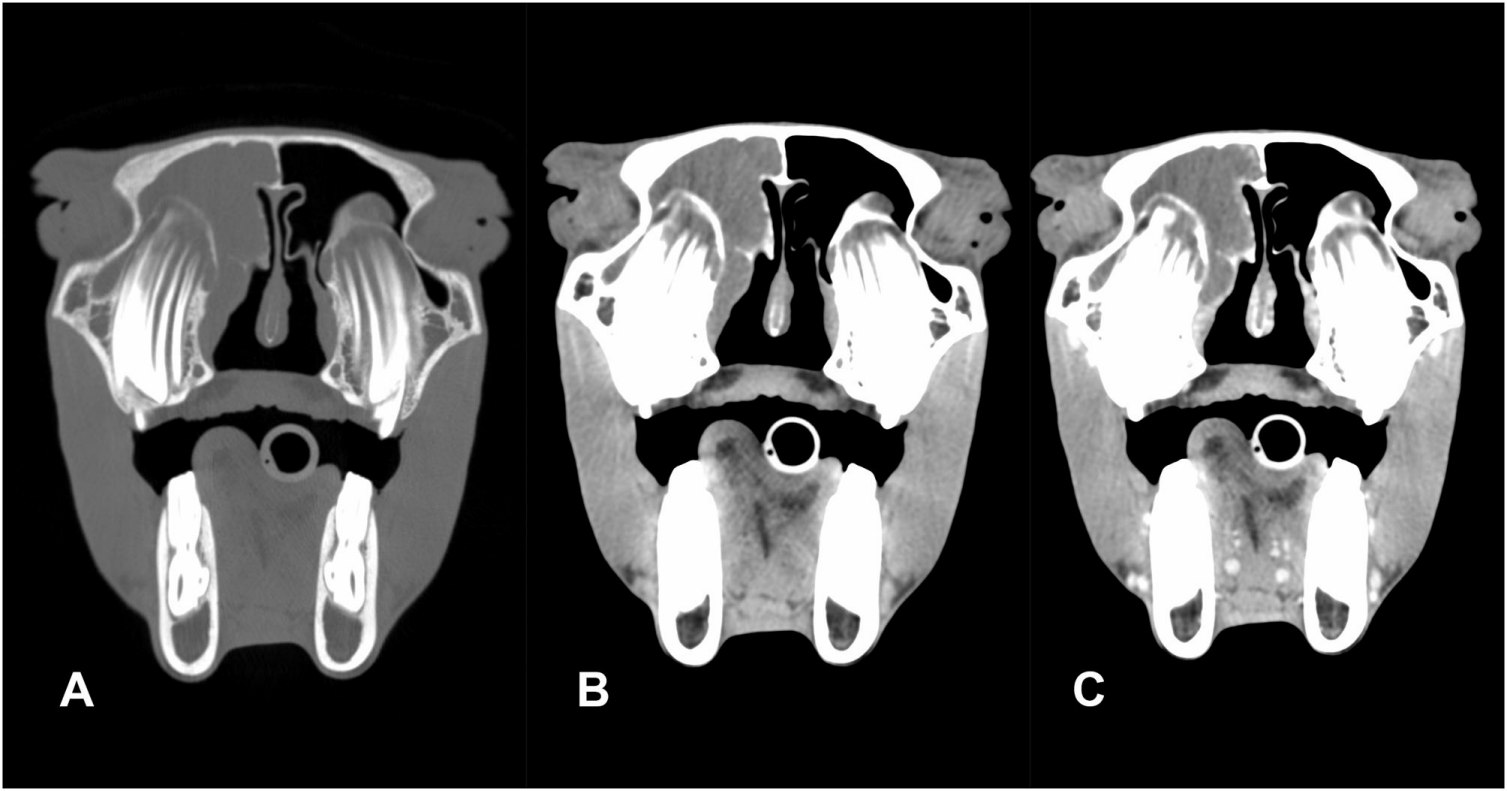
Transverse pre-contrast CT images of an American miniature horse with unilateral disease (case 5) processed with bone **(A)** and soft tissue **(B)** filters and post-contrast CT image **(C)** (left in the image is right of the patient): the right sinus compartments were diffusely filled with fluid attenuating material (HU: 20). There is subtle leftward deviation of the nasal septum. There is mild contrast enhancement of the mucosal lining of the concho-frontal sinus but no enhancement of the sinus content.

A post-contrast study was performed in five of six patients (cases 1–5); in none of these was contrast enhancement of the sinus content detected. In three patients (cases 3–5), rim enhancement was noted surrounding the content representing most likely the mucosal lining of the affected sinus compartment (Figure 3). In five patients, the medial retropharyngeal lymph nodes were included in the scan. In three of these (cases 2, 3, and 7), mild to moderate lymphadenopathy was present. None of the patients underwent a follow-up CT study.

**Figure 4:**
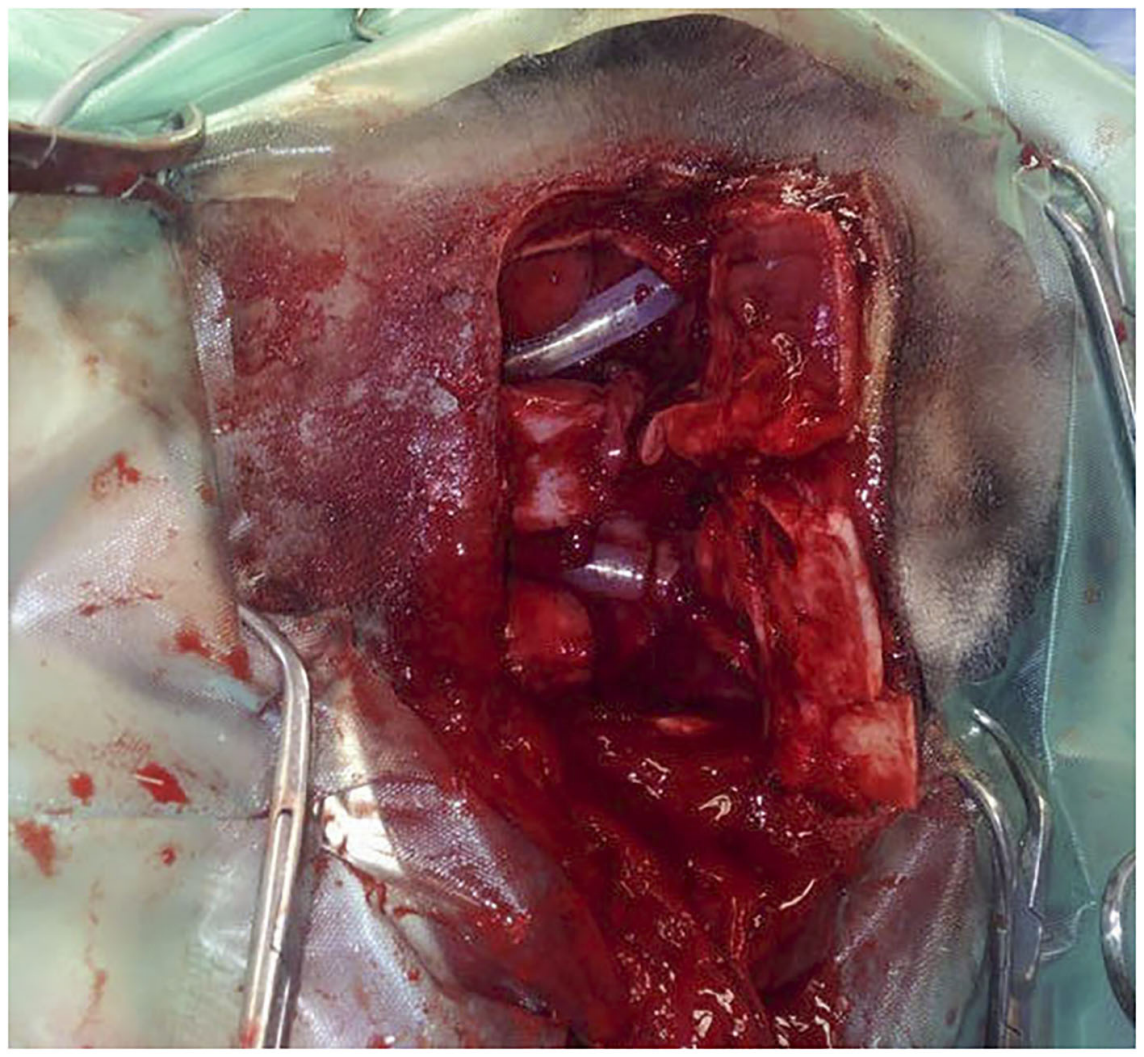
Intra-operative dorsal view of a bilateral fronto-nasal bone flap osteotomy in a Miniature horse (case 3) with obstructive disease of the sino-nasal passages. Animal in left lateral recumbency. The bone flap is opened and silicone tubes have been introduced in both nasal passages after perforation of the medioventral wall of the dorsal concha.

Subsequent surgical treatment was straightforward in all cases. Five horses (cases 1–4 and 7) were treated under general anesthesia. Horses were given acepromazine (0.03 mg/kg) intramuscularly 30 min prior to surgery. Premedication included romifidine (0.2 mg/kg) and morphine (0.1 mg/kg) intravenously. Induction of anesthesia was done by intravenous injection of ketamine (0.2 mg/kg) and midazolam/diazepam (0.15 mg/kg). Following oro-tracheal intubation in four of five horses, maintenance of anesthesia was done with a mixture of oxygen and isoflurane. The tracheotomy performed pre-operatively in case 2 was used for intubation instead of oro-tracheal intubation. Surgery was performed in the two other horses (cases 5 and 6), conscious and standing under sedation protocols. Intravenous premedication included detomidine (0.01 mg/kg) and methadone (0.5 mg/kg). The appropriate plane of sedation was maintained using a CRI of detomidine (0.01 mg/kg/h). Local analgesia was provided by regional anesthesia with mepivacaine (bilateral maxillary nerve perineural analgesia; subcutaneous infiltration along the surgical incision lines). When opening the sinus compartments, large volumes of mucus exuded and were evacuated using suction. The right concho-frontal and caudal maxillary sinus compartments of case 2 that had prior trephination and flushing were filled with malodorous, purulent liquid contents. Initially, at the time of trephination, these compartments contained a clear non-purulent fluid. Bacteriological culture was performed in two cases, which resulted in negative outcomes. No bacteriological culture was performed of the contents from the horse that had prior trephination. Cytology was not performed in any of the cases. Exploration of the emptied sinuses did not reveal other pathological conditions in all animals. Profuse bleeding following perforation of the dorsal conchal sinus floor necessitated packing the sinus compartment with gauze bandages in case 1. In all other cases, minimal bleeding occurred and was controlled using temporary pressure with gauze sponges. Silicone tubes (*n* = 5) or Foley catheters (*n* = 1) were introduced into the affected nasal passages and fixed (Figure 4). In case 3 with bilateral sinus disease, a tracheotomy was performed to facilitate breathing before recovery from anesthesia. Mean surgery time was 64 min (±17).

Premature loss of tubes/Foley catheters was recorded in three cases after 3 (case 1), 5 (case 7), and 6 days (case 2), respectively. No attempts were made to replace these. In two other cases (4 and 5), tube fixation at the level of the nostrils was observed to loosen after 2 days and 2 weeks, respectively, which was subsequently restored by replacing the fixation sutures. A period of 3 weeks during which tubes could be kept in place was achieved in four of seven cases. Wound healing of sinusotomy incisions was straightforward in all cases. A pre-operative existing local wound infection present at the right trephination site in case 2 required daily post-operative wound care consisting of regular (two to three times per day) disinfection (chlorhexidine 0.1%) and application of a chlorhexidine diacetate containing wound ointment. Swelling and drainage of pus resolved within 3 weeks. Post-operative endoscopy was performed in three of seven animals (cases 2, 4, and 6) at varying time intervals (3, 4, and 8 weeks post-op respectively). These examinations showed complete resolution of obstruction of the nasal passages and did not reveal any anomalies at the level of the natural apertures. The surgical fenestration was visualized and still patent in two of these animals (cases 2 and 6). Both had experienced premature loss of the draining tubes.

Radiographic follow-up was available for cases 2 and 5; in both patients, there was resolution of the soft tissue opacity of the affected sinus compartments. Both patients demonstrated mild to moderate bony changes such as periosteal reaction, thickening, and mild deformation of the frontal bone related to the surgery site.

Long-term outcome following a median period of 19 months was very good in all cases. Occasional mucus discharge was reported in two of seven cases (2 and 7). Cosmetic outcome was considered good in six of seven cases; only one case (1) reported a small permanent bump at the level of the surgical incision. Respiratory noise when running was reported in two of seven cases (3 and 7) without interfering with performance.

## Discussion

Idiopathic sino-nasal obstruction is very rarely encountered in horses. This is reflected in veterinary literature by the publication of only two papers over six decades addressing this problem, which, based on our results, seems to be specifically associated with miniature horse breeds of younger ages (12, 13). Common causes for occlusion of sino-nasal communication such as chronic infection, trauma, or previous surgery were all excluded in these cases. Underlying allergic causes were not examined preoperatively but the absence of continued nasal discharge following surgical treatment in the majority of animals also makes this very unlikely. The bilateral presentation of the problem in six of seven animals at a very young age suggests a developmental origin and possible breed predisposition. Unfortunately, preoperative endoscopic evaluation of the nasomaxillary aperture was not possible in most cases as the endoscope could not be advanced sufficiently into the nasal passages. Post-operative endoscopy performed in three of seven animals at varying time intervals did not reveal anomalies at the level of the natural apertures. The presence of large reserve dental crowns disproportionate to the size of these animals' skulls (that is common in small breeds) might have also contributed to an obstructive effect on normal nasomaxillary clearance mechanisms. Complete obstruction of any sino-nasal communication was only encountered in one animal with no nasal discharge whose signs progressed to severe respiratory airflow obstruction characterized by open mouth breathing. This illustrated the potentially progressive nature of the disease. Initial conservative treatments by administration of antibiotics and/or NSAIDs were unsuccessful in all animals.

A comparable syndrome to that described in the present paper, termed “sinus mucocele,” was previously reported in a 6-year-old American miniature horse (13). An older report (12) mentioned treatment of a unilateral accumulation of fluid in the sinus, which was called “mucocele sinusitis.” In humans, confusing descriptions of sinus mucocele have been reported including “cyst-like” as well as “thick-walled” structures (16). The lining is reported to be mucoperiosteum (17) or respiratory epithelium (18), and their location has been reported to be under the periosteum, which explains their potentially destructive effects due to expansile properties that cause erosion and remodeling of surrounding bony structures (17). None of these characteristics have been recorded in the present equine study where a mere accumulation of normal secretion from the sinus respiratory mucosa within the predefined sinus cavities was found. Although this accumulation caused important distortion of the inner sinus outlines, external bony deformations were only observed in one animal with focal outward bulging of the frontal bone and thinning of the bone due to pressure osteolysis. In equine paranasal sinus cysts, the presence of osteoclastic giant cells is thought to be responsible for the remodeling because of distension fluid pressure resulting in commonly reported facial swellings (18). Deformation of the skull as was observed in a second animal was related to infection of a former trephination site presenting with periosteal reactions and subcutaneous abscess formation. Because of these discrepancies, the authors preferred not to use the term “mucocele” for the encountered pathology.

The imaging features recorded in the present study have some similarities with the appearance of paranasal sinus cysts. Paranasal sinus cysts are commonly unilateral; bilateral disease is only rarely reported. In a recent study (19) investigating CT characteristics of paranasal sinus cysts, seven of eight cysts demonstrated a soft tissue to mineral attenuating wall; this finding was not detected in this case series of sino-nasal obstruction. Paranasal sinus cysts are typically expansile lesions; therefore, bone distortion is a common finding. In one patient in our case series, a true distortion of the frontal bone was present. This was on the side of the most severely affected sinus complex, and marked septal deviation toward the contralateral less affected side was also present. Duration of the disease in this patient was not significantly longer than in the other patients of this case series. Varying degrees of septal deviation away from the most severely affected sinus complex was a common finding, indicating that fluid accumulation associated with this pathology can increase pressure on the affected side. Primary sinusitis also presents as diffuse fluid accumulation in the affected sinus complex, which is purulent. Primary sinusitis is, however, rarely bilateral. Recorded HU of the sinus content varied from 1 to 25 in this case series. In one patient, the measured HU values were slightly higher; however, during surgery, the fluid content in this patient did not have different characteristics compared to the other patients. The CT study of this patient demonstrated some streaking artifacts, which could be responsible for this difference. In the patient with secondary infection following trephination, the recorded values did not differ from those measured in sinus compartments filled with clear fluid. A recent study demonstrated that HU measurements are not reliable to differentiate between cyst fluid and sinus mucus or exudate (19). Recorded HU were in the same range as those measured in the present study excluding HU as a means of differentiating between primary sinusitis and the idiopathic pathology in this case series.

The treatment performed included surgical sinus access through a large fronto-nasal bone flap osteotomy and perforating the conchal wall for re-establishment of more permanent drainage to the nose proved to be successful for all cases without recurrence of fluid accumulation. However, a more minimally invasive approach, such as transendoscopic laser fenestration (20, 21) or endoscopy-guided transnasal conchotomy (22, 23), might be more sufficient to restore sino-nasal drainage in these cases. However, the obstructed nasal passages precluded such an approach. The surgical fenestrations that were created in the present case series were large enough to stay patent in the long term as was confirmed endoscopically in two patients or remained at least patent until re-establishment of the natural aperture following age-related changes in the relationship between teeth and sinus structures. Patency of surgical fenestrations was not negatively influenced by premature tube loss, which questions the necessity of using these tubes for this purpose. The size of the fenestrations is most likely more important to maintain patency in the long term. Re-examining treated animals with endoscopy would enable to make more definite conclusions.

Surgical fenestration in the medioventral wall of the ventral conchal sinus (VCS) would have facilitated greater fluid loss than the more dorsal position used in this case series. However, the floor of the dorsal conchal sinus was preferred as the perforation site because of easiness of access to this region compared to the VCS. The presence of large reserve crowns in these young animals precluded access or only allowed blinded access to the VCS. The chances of causing important bleeding in a location that was difficult to access were considered much higher and thus not preferred. Use of a recently reported digital depression technique could have prevented hemorrhage such as was encountered in one case and might have resulted in adequate drainage in a more ventral position than that reported in the present case series (24).

Long-term outcome following a surgical fronto-nasal sinusotomy approach and nasomaxillary drainage repair for treatment of sino-nasal obstruction in miniature pony breeds achieved satisfactory outcomes with acceptable complications. Miniature equine breeds seem to be susceptible to the development of this rarely encountered sinus pathology and are particularly vulnerable at an age range where the developing dentition causes additional compression of the nasal passages. This should be considered in the differential diagnosis of progressive facial deformation and associated respiratory noises in these breeds.

## Data Availability Statement

The raw data supporting the conclusions of this article will be made available by the authors, without undue reservation.

## Ethics Statement

Ethical review and approval was not required for the animal study because this is a retrospective study that didn't include any experimental aspects. Written informed consent for participation was not obtained from the owners because the study was a retrospective study where the research was initiated months after surgical correction of a disease problem was done. All owners were contacted by telephone inquire and questioned about participating to the study. They all agreed and provided the necessary information for the follow-up part of the study.

## Author Contributions

LV was responsible for conceptualization, writing (original draft preparation), and editing. ER was responsible for writing (original draft preparation) and editing. All other authors contributed to reviewing and editing.

## Conflict of Interest

The authors declare that the research was conducted in the absence of any commercial or financial relationships that could be construed as a potential conflict of interest.

## References

[1] O'Leary JM Dixon PM. A review of equine paranasal sinusitis: aetiopathogenesis, clinical signs and ancillary diagnostic techniques. Equine Vet Educ. (2011) 23:148–59. 10.1111/j.2042-3292.2010.00176.x

[2] Dixon PM O'Leary JM. A review of equine paranasal sinusitis: medical and surgical treatments. Equine Vet Educ. (2011) 24:143–58. 10.1111/j.2042-3292.2011.00245.x

[3] Dixon PM Parkin TD Collins N Hawkes C Townsend N Tremaine WH. Equine paranasal sinus disease: a long-term study of 200 cases (1997-2009): ancillary diagnostic findings and involvement of the various sinus compartiments. Equine Vet J. (2012) 44:267–71. 10.1111/j.2042-3306.2011.00420.x 21812807

[4] Woodford NS Lane JG. Long-term retrospective study of 52 horses with sinunasal cysts. Equine Vet J. (2006) 38:198–202. 10.2746/042516406776866372 16706271

[5] Rothaug PG Tulleners EP. Neodymium-yttrium-aluminum-garnet laser-assisted excision of progressive ethmoid hematomas in horses: 20 cases (1986-1996). J Am Vet Med Assoc. (1999) 214:1073–41. 10200800

[6] Dixon PM Parkin TD Collins N Hawkes C Townsend NB Fisher G. Historical and clinical features of 200 cases of equine sinus disease. Vet Rec. (2011) 169:439–43. 10.1136/vr.d4844 21868434

[7] Head KW Dixon PM. Equine nasal and paranasal sinus tumours. Part 1: review of literature and tumour classification. Vet J. (1999) 157:261–78. 10.1053/tvjl.1998.0370 10328838

[8] Dixon PM Head KW. Equine nasal and paranasal sinus tumours. Part 2: a contribution of 28 case reports. Vet J. (1999) 157:279–94. 10.1053/tvjl.1999.0371 10328839

[9] Marks SC Latoni JD Mathog RH. Mucoceles of the maxillary sinus. Otolaryngol Head Neck Surg. (1997) 117:18–21. 10.1016/S0194-5998(97)70200-6 9230317

[10] Busaba NY Salman SD. Maxillary sinus mucoceles: clinical presentation and long-term results of endoscopic surgical treatment. Laryngoscope. (1999) 109:1446–9. 10.1097/00005537-199909000-00017 10499053

[11] Tremaine WH Dixon PM. A long-term study of 277 cases of equine sinonasaal disease. Part 1: details of horses, historical, clinical and ancillary diagnostic findings. Equine Vet J. (2001) 33:274–82. 10.2746/042516401776249615 11352350

[12] Hensley RM Thomas EW. Mucocele sinusitis in a thoroughbred filly. J Am Vet Med Assoc. (1957) 130:133–4. 13416075

[13] Easley JT Freeman DE. A single caudally based frontonasal bone flap for treatment of bilateral mucocele in the paranasal sinuses of an American Miniature horse. Vet Surg. (2013) 42:427–32. 10.1111/j.1532-950X.2013.01093.x 23373723

[14] Freeman DE Orsini PG Ross MW Madison JB. A large frontonasal bone flap for sinus surgery in the horse. Vet Surg. (1990) 19:122–30. 10.1111/j.1532-950X.1990.tb01152.x 2333683

[15] Staszyk C Bienert A Bäumer W Feige K Gasse H. Simulation of local anaesthetic nerve block of the infraorbital nerve within the pterygopalatine fossa: anatomical landmarks defined by computed tomography. Res Vet Sci. (2008) 85:399–406. 10.1016/j.rvsc.2008.02.008 18371997

[16] Caylakli F Yavuz H Cagici AC Ozluoglu LN. Endoscopic sinus surgery for maxillary sinus mucoceles. Head Face Med. (2006) 2:29. 10.1186/1746-160X-2-29 16953897 PMC1570343

[17] Skoulakis CE Velegrakis VA Doxas PG Papadakis CE Bizakis JG Helidonis ES. Mucocele of the maxillary antrum in an eight-year-old boy. Int J Pediatr Otorhinolaryngol. (1999) 47:283–87. 10.1016/S0165-5876(99)00002-6 10321785

[18] Tremaine WH Clarke CJ Dixon PM. Histopathological findings in equine sinonasal disorders. Equine Vet J. (1999) 31:296–303. 10.1111/j.2042-3306.1999.tb03820.x 10454087

[19] Ostrowksa J Lindström L Toth T Hansson K Uhlhorn M Ley CJ. Computed tomography characteristics of equine paranasal sinus cysts. Equine Vet J. (2020) 52:538–46. 10.1111/evj.13212 31793020

[20] Kolos F Bodecek S Zert Z. Trans-endoscopic diode laser fenestration of equine conchae *via* contralateral nostril approach. Vet Surg. (2017) 46:915–24. 10.1111/vsu.12680 28643340

[21] Morello SL Parente EJ. Laser vaporization of the dorsal turbinate as an alternative method of accessing and evaluating the paranasal sinuses. Vet Surg. (2010) 39:891–9. 10.1111/j.1532-950X.2010.00728.x 20723190

[22] Bach FS Böhler A Schieder K Handschuh S Simhofer H. Surgical enlargement of the nasomaxillary aperture and transnasal conchotomy of the ventral conchal sinus: two surgical techniques to improve sinus drainage in horses. Vet Surg. (2019) 48:1019–31. 10.1111/vsu.13207 30968454 PMC6767416

[23] Zukin LM Hink EM Liao S Getz AE Kingdom TT Ramakrishnan VR. Endoscopic management of paranasal sinus mucoceles: meta-analysis of visual outcomes. Otolaryngol Head Neck Surg. (2017) 157:760–6. 10.1177/0194599817717674 28695766 PMC5665697

[24] Carmalt JL. Intraoperative depression of the bulla of the maxillary septum as a method of improving sinus drainage without epistaxis in horses. Eq Vet Educ. (2020). 10.1111/eve.13402. [Epub ahead of print].

